# Minimal exposure technique in zygomatic implants: A case report

**DOI:** 10.6026/973206300213456

**Published:** 2025-10-31

**Authors:** Divye Malhotra, Variyta V, Shubh Karmanjit Singh Bawa, Parul Sharma, Akshat Sharma

**Affiliations:** 1 Department of Oral and Maxillofacial Surgery, Himachal Dental College, Sundernagar, District Mandi, Himachal Pradesh, India; 2 Department of Oral and Maxillofacial surgery, Baba Farid University of Health Sciences, Sadiq Road, Faridkot, Punjab, India; 3 Department of Periodontolgy and Implantology, Himachal Dental College, Sundernagar, District Mandi, Himachal Pradesh, India

**Keywords:** Zygomatic implants, minimal exposure technique, atrophic maxilla, flapless surgery, maxillary rehabilitation

## Abstract

The Minimal Exposure Technique for Zygomatic Implants (METZI) is a graft-free method for patients with severe upper jaw bone loss. It minimizes surgical trauma and risk of paresthesia while ensuring stable implant integration. The technique involves detailed pre-surgical planning and vestibular incisions for precise implant placement with minimal tissue invasion. METZI has shown improved implant stability and reduced complications such as hematoma, mucositis and oroantral communication. Overall, METZI offers a safe, less invasive solution for maxillary rehabilitation with favorable outcomes.

## Background:

Zygomatic implants have changed maxillary re-building through the provision of a reliable, graft-free option where provisional implant placement is precluded from severe posterior maxillary atrophy, excessive pneumatized sinuses or when previous grafting was unsuccessful [1]. First developed in the second half of the 20th century, these long implants provide fixture engagement with dense zygomatic bone, reconciling engagement with the zygomatic bone in addition to the maxillary alveolus, permitting implant support in a cortical site, with more primary stability than the compromised posterior maxilla alone. Zygomatic implants avoid the need for extensive bone grafting and two-staged augmentation of the posterior maxilla and therefore shorten the overall treatment timing and broaden treatment options for patients that would require grafting, augmented staged procedures requiring surgery with the associated risks and time [2]. Indications for zygomatic implants range from extreme vertical/or horizontal atrophy of the posterior, failed sinus augmentations, post-oncological resections where residual native bone was insufficient, or in consideration of patients' decisions to avoid grafting process which is inherently a two-staged event. Contraindications primarily relate to local anatomy or active pathology (active sinus infection, inadequate zygomatic anatomy, or medical condition contraindicating surgery) [3]. Consideration of these factors is critical for appropriate case selection and consideration needs to include, consideration of the evaluation of zygomatic bone evaluation (volume and quality), health and quality of the maxillary sinus, soft-tissue biotype, occlusal scheme or parafunction and realistic end point for prosthetics [4]. Zygomatic implant placement has an established set of surgical techniques that include several intrasinus and extrasinus techniques, with sinus-slot and guided/flapless techniques being categorized under either approach. The intrasinus (classic) technique places the implant through or below the sinus wall into the zygoma; the extrasinus and sinus-slot techniques aim to minimize sinus membrane manipulation and exposure by placing the implant more laterally or creating a slot to preserve or minimize contact with the Schneiderian membrane. Each approach is tailored to provide balance between access, visibility and potential for sinus related complications with the final selection dictated by the individual anatomical site and surgeon preference [5]. Flapless and minimally invasive approaches (for example, Minimal Exposure Technique (MET) and vestibular minimal-access incisions) are performed to potentially reduce trauma to the soft tissue while preserving blood supply, reducing healing time and enhancing postoperative comfort. These approaches require meticulous planning before surgery and a high level of accuracy during osteotomy and implant insertion to avoid soft-tissue damage because the defined and limited exposure reduces adjustment intraoperatively which is often needed with a less defined surgical approach. The attractions of these approaches is potentially less morbidity, swelling and pain postoperatively and quicker return to function, assuming clear and accurate surgical execution of the plan [6]. The accurate preparation of an osteotomy and proper implant placement are essential to the success of zygomatic implants, especially when considering immediate loading. Although immediate loading may be advantageous for both the patient and the clinician, it does depend on verifying sufficient primary stability and providing a suitable biomechanical environment for provisionalization, for example, immediate loading could afford advantages in treatment time, time until restoration of masticatory function, esthetics, patient satisfaction and avoid a removable interim prosthesis. Wherever applicable, the primary stability needed along the length of a zygomatic implant can only be achieved with precise angulation and depth control while maximizing cortical engagement of the zygoma and protecting adjacent anatomic structures [7].

Piezoelectric (piezo) bone surgery provides a device-level answer to many surgical precision issues. Piezoelectric instruments use micro-vibrations at ultrasonic frequencies to selectively cut mineralized tissue while sparing soft tissues. When used for the preparation of zygomatic implant sites, the advantages of piezo surgery include: precise, micrometric bone cutting with reduced risk of harming the sinus membrane, neural bundles, or soft tissues; less thermal necrosis with continuous irrigation; excellent tactile sensitivity that enables the surgeon to feel the quality of bone and cortical thickness; and good visibility as the cavitational effect and irrigation subsides intraoperative bleeding [8]. All of these qualities are particularly useful when performing flapless and minimal access techniques where very little exposure elevates the need for making accurate and safe cuts. With the ability to carry out precise osteotomies while respecting the anatomy of the sinus, while preserving soft-tissue integrity, piezoelectric surgery could reduce intraoperative complications (membrane perforation), postoperative morbidity (pain, swelling, sinusitis) and can enhance the reliability of achieving primary stability of the implant [9]. In addition to the advantages of surgical accuracy, combining piezoelectric techniques with new imaging and planning technologies cone beam CT (CBCT), virtual surgical planning and patient specific guides-can enhance implant trajectories and limit intraoperative guesswork. Preoperative CBCT imaging allows three-dimensional assessment of zygomatic bone anatomy, sinus pneumatization and relation to other structures, virtual planning can plot ideal implant paths and prospective locations of prosthetic framework support and surgical guides assume the virtual plan is successfully integrated into the operative field. In indirect or flapless cases, thorough preoperative planning combined with piezo-mechanical osteotomy techniques can provide the surgeon the necessary tools to accomplish the plan in a reproducible fashion [10]. Prosthetic considerations are also important: zygomatic implants are also often used in the completion of cross-arch fixed full-arch restorations and the prosthesis must consider implant angulation, framework rigidity, occlusal load distribution and hygiene access. Inside of the order of immediate extraction, the provisional prosthesis needs careful consideration and design to allow it to splint the implants and distribute forces protecting the osteotomy-implant interface while allowing for initial healing. Long term prosthetic success depends on passive fit of the superstructure, the correct occlusal scheme and a maintenance protocol designed to minimize the risk of peri-implantitis all factors that relate to the original surgical accuracy [11].

Zygomatic implant complications include sinusitis, oroantral communications, soft tissue dehiscence, paresthesia, malposition and unfavorable prosthetic emergence and, infrequently, implant failure. The incidence and severity of any of these complications is a function of specific surgical technique and skill level, osteotomy, sinus approach and management and postoperative care. Thus, methods of reducing trauma to the sinus membrane and the surrounding soft tissue, such as sinus-sparing techniques and accurate osteotomy tools, such as piezoelectric surgery, are appealing in mitigating complication rates [12]. While growing clinical series and systematic reviews have reported favorable survival and success rates of zygomatic implants in the hands of experienced surgeons, the literature also highlights the technique-sensitive nature of the procedure and describes the need for appropriate training and standardized protocols. In an effort to minimize morbidity and enhance patient-oriented outcomes, comparative studies suggest that minimally invasive approaches can be utilized in careful planning and protocols for zygomatic implants, but they do rely on strong intraoperative control over osteotomy management and implant placement, which is a method that could be supported by piezoelectric surgery [13]. The development of zygomatic implantology methods towards flapless/minimally invasive modalities, immediate loading and incorporation with digital planning all underscore the importance of surgical accuracy, soft-tissue preservation and predictable primary stability. Piezoelectric surgery addresses many of these priorities by allowing for selective, controlled bone cuts with less risk of damage to adjacent soft-tissue and is therefore a logical adjunct to current zygomatic implant operational protocols [14]. Therefore, it is of interest to report the use of piezoelectric surgery methods to achieve precise zygomatic implant placement with immediate loading and the effects this strategy has on implant stability and surgical morbidity and patients' perceptions of their experiences.

## Patient and observation:

## Patient information:

This was a case of a 46 -year-old female, who needed prosthetic rehabilitation for her jaw-related deformities but opted out of further bone graft procedure. In this case, the procedure was performed under general anesthesia via nasotracheal intubation. Three upper incisors were extracted followed by one zygomatic and three standard implants.

## Clinical findings:

Minimal tissue stress and precision implant placement was ensured.

## Timeline of current episode:

[1] Initial consultation and image evaluation

[2] Planning surgery using the MET-ZI approach

[3] Implant implantation and subsequent care.

[4] Follow-up evaluations for implant stability and healing.

## Therapeutic interventions:

Zygomatic implant placement procedure is started by palpating the infra zygomatic crest and selecting a point 3-4 mm anterior to the zygomatic-maxillary buttress. After this, a 2-3 cm vestibular incision is made vertically, which is followed by carefully sub-periosteal tissue elevation, exposing the zygomatic omaxillary complex. Piezosurgery was used to expose the Schneiderian membrane with minimal disruption. Control drilling through the external cortical layer of the zygomatic bone. Implant fixtures were aligned with the alveolar crest and secured with sutures. A 5 mm tissue punch was used to create a mucosal incision which is the intraoral emergence point in the canine-premolar region. This punch is followed by carefully controlled drilling through the external cortical of the zygomatic bone. The emergence of the drill end is checked using textile sensation in the periorbital region. The drilling procedure is finished by assessing the sinus membrane using a Valsalva maneuver, where the absence of air bubbles confirms the intact membrane. For the optimal prosthetic alignment, the emergence of the implant fixture should ideally be at the alveolar crest. Before the final fixation of the abutment, the angle is adjusted for prosthetic compatibility. The whole procedure is concluded by placing the sutures.

## Postoperative care:

The patient after surgery was put on a five-day regimen of amoxicillin with clavulanic acid (625mg) and NSAID (paracetamol-650 mg) thrice a day for three consecutive days. The patient was also prescribed nasal saline spray along with 0.2 % chlorhexidine oral rinse. Post-operative instructions about strictly maintaining oral hygiene and avoiding tobacco, nose blowing and rigorous rinsing of mouth for ten days was explained to the patient.

## Follow-up and outcome of interventions:

Implant stability: Excellent osseointegration observed in the OPG.

Healing: Minimal swelling and no significant complications reported.

Patient satisfaction: High due to reduced postoperative discomfort and early prosthetic loading.

## Patient perspective:

Patient expressed satisfaction with the procedure, highlighting reduced discomfort and quick recovery. She appreciated the minimally invasive approach and immediate functional benefits of the implants.

## Informed consent:

Informed consent was obtained from patient before the procedure, ensuring understanding of risks and benefits.

## Discussion:

The flapless zygomatic implants have changed the missing teeth rehabilitation for atrophic maxillaby introducing a grafless and minimally invasive as an alternative. This technique, unlike the conventional implant methods, does not need extensive grafting and anchors the implants in zygomatic bone, providing stability in even severe bone loss cases [15]. In comparison to longer treatment time cases like sinus augmentation, short or tilted implants zygomatic implants, zygomatic implants offer a better solution for missing teeth rehabilitation where the bone is atrophic in the maxilla [16]. These challenges were addressed by the MET-ZI method, which enhances visibility while decreasing tissue trauma. By engaging in the vestibular incision, MET-ZI ensures precise implant placement and decreases postoperative complications such as numbness, bruising, inflammation, sinus communication, implant exposure and infections [17]. The researchers have suggested the possible links of post-zygomatic implant paresthesia to infraorbital and zygomaticofacial nerve damage, with studies reporting incidences of 5.4 % (Besdrosdian) and 4.6% (Aparicioetal). MET-ZI's minimally invasive nature significantly lowers these risks [18]. Hematomas occur in about 4% of cases, posing a biological risk to adjacent structures. However, with MET-ZI, the incidence of hematoma is reduced, enhancing surgical safety. In the same manner, Schneiderian membrane perforation and oral-antralfistula (OAF) occur in1.5%- 29% of cases. MET-ZI's improved visibility and enhances membrane repair potential, reducing OAF risk. The studies have confirmed that membrane perforation does not affect survival rates, with a 97.68% survival rate in cases where implants are placed under repaired membranes [19]. The technique coincides with modern trends in patient recovery and low post-treatment traumas. The MET-ZI method also has decreased post-surgical swelling and bruising by improving bone-implant contact [20]. This procedure also aims to reduce sinus exposure. Due to its minimal invasiveness, patients tend to experience lower discomfort compared to traditional zygomatic implant techniques. Another main benefit is immediate loading, which allows for provisional prostheses soon after surgery. This is unlike grafting techniques, which require delayed prosthetic placement. The vestibular suture line improves impression accuracy and reduces the risk of wound dehiscence.

## Conclusion:

Flapless zygomatic implants provide a minimally invasive, cost-effective and efficient solution for rehabilitating the atrophic maxilla. They offer success rates comparable to traditional techniques with improved patient outcomes. As oral implantology advances, their use is expected to expand in managing severe bone loss.

## Competing interests:

We have no conflicts of interest.

## Authors' contributions:

Acquisition of data, study concept and design - Dr. Divye Malhotra

First draft was written by Dr. Variyta and all authors commented on that.

Final manuscript was edited by Dr. Shubh Karman and Dr. Parul and supervised the entire finding of the work.

Dr. Akshat acquired the observation and oversaw the entire project.
